# Cytotoxic Effects of Darinaparsin, a Novel Organic Arsenical, against Human Leukemia Cells

**DOI:** 10.3390/ijms24032282

**Published:** 2023-01-23

**Authors:** Bo Yuan, Hidetomo Kikuchi, Jingmei Li, Atsushi Kawabata, Kozo Yao, Norio Takagi, Mari Okazaki

**Affiliations:** 1 Laboratory of Pharmacology, Graduate School of Pharmaceutical Sciences, Josai University, Sakado 350-0295, Japan; 2 Laboratory of Pharmacotherapy, Graduate School of Pharmaceutical Sciences, Josai University, Sakado 350-0295, Japan; 3 Product Development Division, Solasia Pharma K.K., Tokyo 105-0011, Japan; 4 Department of Applied Biochemistry, School of Pharmacy, Tokyo University of Pharmacy & Life Sciences, Hachioji 192-0392, Japan

**Keywords:** darinaparsin, arsenite, leukemia cells, cell death, cell cycle arrest, p53, c-Myc, notch1 signaling

## Abstract

To explore the molecular mechanisms of action underlying the antileukemia activities of darinaparsin, an organic arsenical approved for the treatment of peripheral T–cell lymphoma in Japan, cytotoxicity of darinaparsin was evaluated in leukemia cell lines NB4, U-937, MOLT-4 and HL-60. Darinaparsin was a more potent cytotoxic than sodium arsenite, and induced apoptosis/necrosis in NB4 and HL-60 cells. In NB4 cells exhibiting the highest susceptibility to darinaparsin, apoptosis induction was accompanied by the activation of caspase-8/-9/-3, a substantial decrease in Bid expression, and was suppressed by Boc-D-FMK, a pancaspase inhibitor, suggesting that darinaparsin triggered a convergence of the extrinsic and intrinsic pathways of apoptosis via Bid truncation. A dramatic increase in the expression level of γH2AX, a DNA damage marker, occurred in parallel with G_2_/M arrest. Activation of p53 and the inhibition of cdc25C/cyclin B1/cdc2 were concomitantly observed in treated cells. Downregulation of c-Myc, along with inactivation of E2F1 associated with the activation of Rb, was observed, suggesting the critical roles of p53 and c-Myc in darinaparsin-mediated G_2_/M arrest. Trolox, an antioxidative reagent, suppressed the apoptosis induction but failed to correct G_2_/M arrest, suggesting that oxidative stress primarily contributed to apoptosis induction. Suppression of Notch1 signaling was also confirmed. Our findings provide novel insights into molecular mechanisms underlying the cytotoxicity of darinaparsin and strong rationale for its new clinical application for patients with different types of cancer.

## 1. Introduction

Despite being a well-known poison, arsenic derivatives have been used medicinally for over 2000 years [1]. Due to its cytotoxicity against various cancer cells, arsenic disulfide has gained increasing attention and been traditionally used to treat certain types of hematological disorders, including acute myeloid leukemia (AML), chronic myeloid leukemia (CML), and myelodysplastic syndrome, in China [2,3,4,5]. Trivalent arsenic derivatives (arsenite, As^III^), such as arsenic trioxide (As_2_O_3_), have also been shown to exhibit high therapeutic efficacy against relapsed and refractory acute promyelocytic leukemia (APL) [1,6,7]. Our interest is in the pharmacokinetics of As_2_O_3_ in clinical samples including peripheral blood, bone marrow and cerebrospinal fluid from APL patients [6,7,8]. Several research groups, including ours, have systematically studied the metabolites of As_2_O_3_ in APL patients, and have further suggested that methylated metabolites of As_2_O_3_ also possess cytocidal activity against leukemia and lymphoma cells [6,7,8,9].

S-dimethylarsino-glutathione, an organic arsenical known as darinaparsin, consists of dimethylarsenic conjugated to glutathione and is one of the metabolites of inorganic arsenic during its biotransformation in the body [10]. Darinaparsin has been demonstrated to be a more potent cytotoxicity inducer than As_2_O_3_ in various malignant cell lines including As_2_O_3_-resistant cancer cells [11,12,13]. Previous studies have demonstrated that darinaparsin has a maximum tolerated dose that is 50-fold higher than As_2_O_3_ in mice [11] and more importantly, that darinaparsin caused no obvious changes in electrocardiographic parameters in patients [14,15] when compared with As_2_O_3_-related QT prolongation. Based on the results of a series of clinical studies [14,15], darinaparsin was approved by the Japanese Ministry of Health, Labour and Welfare in June 2022 for the treatment of relapsed and refractory peripheral T-cell lymphoma (PTCL) [16], which represents a rare and heterogeneous group of clinically aggressive diseases with a poor response to conventional therapy. Despite this, the cytocidal effect of darinaparsin against leukemia cells and its underlying molecular mechanisms remain largely unexplored.

Given a strong link between the dysregulation of apoptosis and cancer development, the targeting of apoptosis has been recognized as a mainstay and a goal of clinical cancer therapy [17,18]. Generally, caspase-8 and -9 are activated during extrinsic and intrinsic apoptotic pathway, respectively, and the extrinsic pathway can converge with intrinsic pathway through the truncation of Bid, a key player in the crosstalk between intrinsic and extrinsic pathways [1,17,19]. In addition, necrosis and cell cycle arrest have been characterized as important cellular processes responsible for the action of many chemotherapeutic drugs [20,21,22,23]. All these cellular processes are closely correlated with the activity of various chemotherapeutic reagents, including arsenic compounds, of inducing DNA damage, which in turn leads to the manipulation of transcription factors such as p53 and c-Myc as well as their respective downstream molecules, and ultimately contributes to cell death and/or cell cycle arrest [24,25]. It is well known that chemotherapeutic reagents can cause various types of DNA damage by virtue of their ability to directly induce single-strand breaks (SSBs)/double-strand breaks (DSBs) and/or oxidative stress [24,25]. Similar to As_2_O_3_, darinaparsin has also been demonstrated to exhibit cytotoxicity against blood cancer cells through the stimulation of oxidative stress [26,27]. Whether and how the abovementioned cellular processes contribute to the cytotoxicity of darinaparsin remains to be seen, as do the key molecules involved in these cellular processes.

In addition, dysregulated Notch1 signaling has been found in numerous cancers, including hematological malignancies [28,29]. A previous report has also demonstrated the multiple mutations of various key players in the regulation of cancer initiation and progression, including Notch1, in PTCL patients [30]. Intriguingly, As_2_O_3_ has been shown to inhibit cell growth and induce apoptosis through inactivation of the Notch1 signaling pathway in different types of cancer cells [31,32]. However, it remains unclear whether darinaparsin impacts the Notch1 signaling pathway and consequently exhibits its cytotoxicity.

In the present study, following the comparison of the cytotoxic effects of darinaparsin and As^III^ against four human leukemia cell lines—NB4, U-937, MOLT-4 and HL-60—induction of apoptosis/necrosis was evaluated in NB4 and HL-60 cells, which exhibited the highest and lowest sensitivity to darinaparsin, respectively. The activation of caspases and Bid truncation was further investigated to clarify how intrinsic and extrinsic pathways contributed to the darinaparsin-mediated apoptosis in NB4 cells. Cell cycle arrest was evaluated by focusing on DNA damage associated with the manipulation of p53 and c-Myc as well as their downstream molecules. Trolox, an antioxidative reagent, was used to investigate the correlation between darinaparsin-triggered oxidative stress and apoptosis as well as cell cycle arrest. The impacts of darinaparsin on Notch1 signaling pathway was also explored.

## 2. Results

### 2.1. More Potent Cytotoxicity Induced by Darinaparsin in Comparison with As^III^ in Human Leukemia Cell Lines

**Figure 1:**
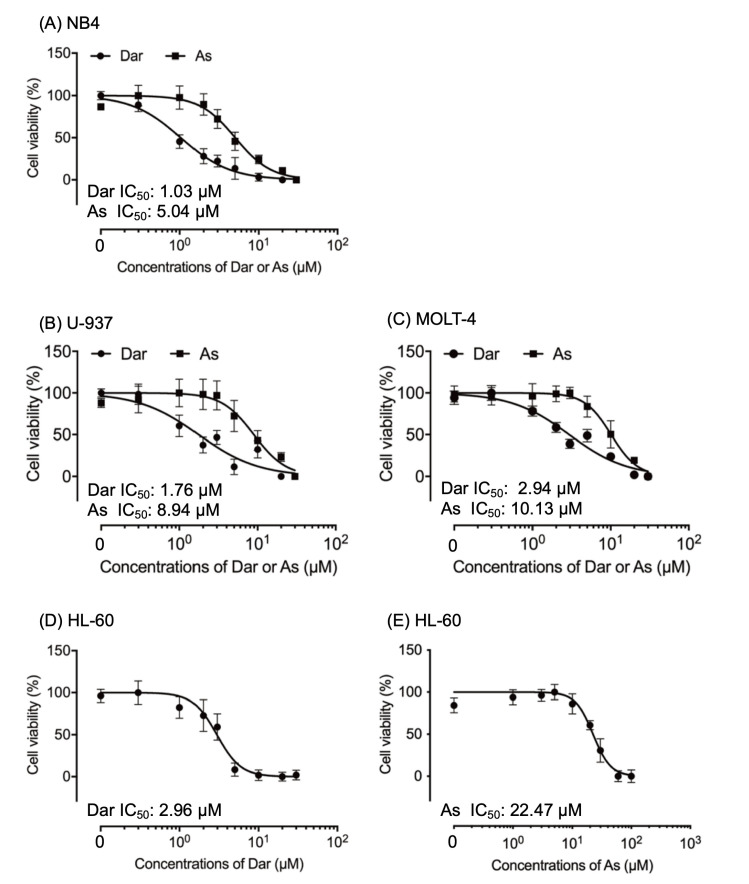
Cytotoxic effects of darinaparsin and As^III^ against human leukemia cell lines. Cell viability of NB4 (**A**), U-937 (**B**) and MOLT-4 (**C**) was determined by WST-1 assay after treatment for 24 h with various concentrations of darinaparsin and As^III^ (0.3, 1, 2, 3, 5, 10, 20, 30 μM). Since HL-60 is more resistant to As^III^ than other cells, its cell viability was determined by WST-1 assay after treatment for 24 h with various concentrations of darinaparsin (0.3, 1, 2, 3, 5, 10, 20, 30 μM) (**D**) and As^III^ (1, 3, 5, 10, 20, 30, 60, 100 μM) (**E**). Relative cell viability was calculated as the ratio of the absorbance at 450 nm of each treatment group against those of the corresponding untreated control group. Data are shown as the means and SD from more than three independent experiments. Dar, darinaparsin; As, As^III^.

A significant decrease in cell viability was observed in a dose-dependent manner in all four leukemia cell lines tested after treatment for 24 h with various concentrations of darinaparsin and As^III^, respectively. As shown in Figure 1, the IC_50_ values of darinaparsin were 1.03 μM (95% confidence interval, 0.97–1.10; R^2^ = 0.9534), 1.76 μM (95% confidence interval, 1.61–1.91; R^2^ = 0.8638), 2.94 μM (95% confidence interval, 2.53–3.41; R^2^ = 0.9326) and 2.96 μM (95% confidence interval, 2.80–3.12; R^2^ = 0.9045) in NB4, U-937, MOLT-4 and HL-60 cells, respectively. Whereas the IC_50_ values of As^III^ were 5.04 μM (95% confidence interval, 4.73–5.38; R^2^ = 0.9229), 8.94 μM (95% confidence interval, 8.16–9.81; R^2^ = 0.8394), 10.13 μM (95% confidence interval, 9.01–11.39; R^2^ = 0.928) and 22.47 μM (95% confidence interval, 20.85–24.11; R^2^ = 0.9257) in NB4, U-937, MOLT-4 and HL-60 cells, respectively. Although the same rank order for the sensitivity to darinaparsin and As^III^: NB4 > U-937 > MOLT-4 > HL-60 was observed, these leukemia cells showed approximately 3–8 times more sensitivity to darinaparsin in comparison with As^III^. The following experiments were thus conducted by focusing on the cytocidal effect of darinaparsin rather than As^III^ against leukemia cells.

### 2.2. Contribution of Apoptosis and Necrosis to the Cytocidal Effect of Darinaparsin against NB4 and HL-60 Cells

**Figure 2:**
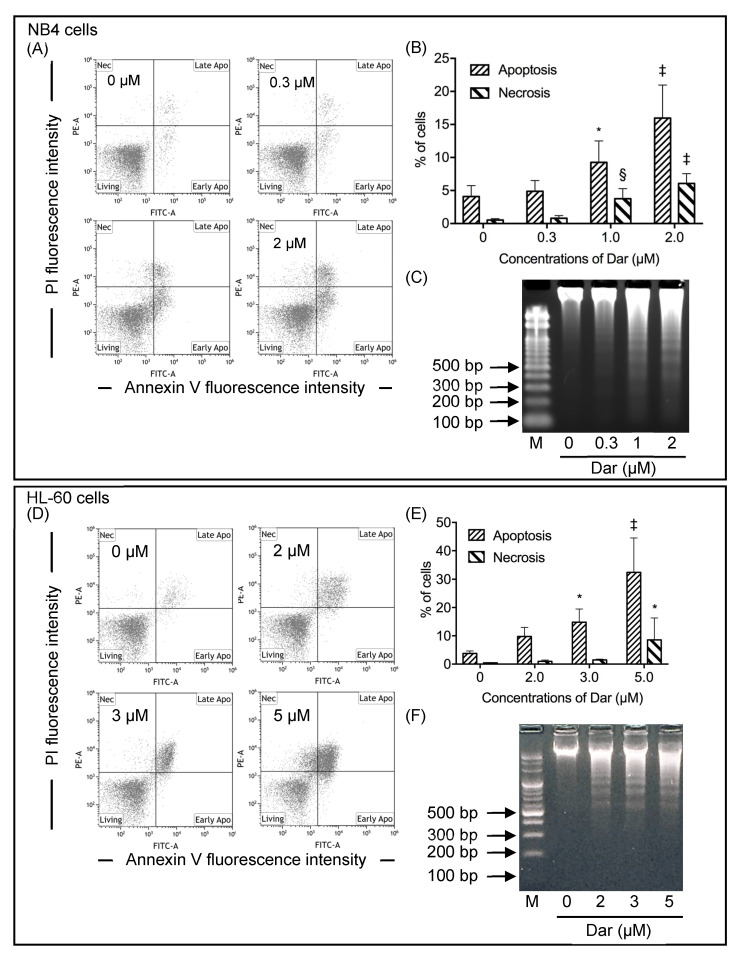
Induction of apoptosis and necrosis in NB4 and HL-60 cells treated with darinaparsin. Determination of apoptosis was conducted following treatment with various concentrations of darinaparsin for NB4 cells (0.3, 1, and 2 μM) (**A**–**C**); and for HL-60 cells (2, 3, and 5 μM) (**D**–**F**), for 24 h. After treatment, cells were stained with annexin V-FITC and PI and analyzed by flow cytometry as described in the “Materials and Methods” section. Annexin V(−)PI(−) cells, annexin V(+)PI(−)/PI(+) cells and annexin V(−)PI(+) cells represent viable cells, apoptotic cells, and necrotic cells, respectively. Representative flow cytometry dot plots from three independent experiments (**A**,**D**), and the quantification of the percentages of apoptotic and necrotic cells (**B**,**E**) were shown. DNA electrophoresis patterns were shown after treatment with indicated concentrations of darinaparsin for 24 h (**C**,**F**). *, *p* < 0.05; §, *p* < 0.001; ‡, *p* < 0.0001 vs. control. Dar, darinaparsin.

Since NB4 and HL-60 cells exhibited the highest and lowest sensitivity to darinaparsin, respectively, among the four leukemia cell lines tested, the underlying mechanisms of the cytocidal effect of darinaparsin against the two representative cell lines were first explored in terms of their induction of apoptosis and necrosis. Based upon the abovementioned IC_50_ values of darinaparsin in the two cells, different concentrations of darinaparsin were determined for the treatment of NB4 and HL-60 cells. First, after exposure of NB4 cells to various concentrations of darinaparsin (0.3, 1, 2 μM) for 24 h, annexin V/PI analysis was conducted to explore whether apoptosis and/or necrosis contributed to the cytotoxic effects of darinaparsin. As shown in Figure 2A,B, treatment with darinaparsin caused the dose-dependent induction of apoptosis in NB4 cells. When the concentration of darinaparsin increased up to 1 μM, approximately equal to its IC_50_ value at 24 h post-exposure, a statistically significant difference was observed between the darinaparsin-exposed group and control group. Along with apoptosis induction, a significant increase in the proportion of necrotic cells was also observed following exposure to darinaparsin at concentrations of 1 or 2 μM. Consistent with annexin V/PI staining results, DNA fragmentation analysis showed that a substantial DNA fragmentation was observed in NB4 cells following treatment with darinaparsin at concentrations of 1 or 2 μM, although only a modest DNA fragmentation was observed after treatment at a concentration of 0.3 μM (Figure 2C). Next, an annexin V/PI analysis was conducted following the exposure of HL-60 cells to various concentrations of darinaparsin (2, 3, and 5 μM) for 24 h. As shown in Figure 2D,E, a dose-dependent apoptosis induction was observed in darinaparsin-treated HL-60 cells. Of note, a statistically significant difference in the proportion of apoptotic cells was observed in cells treated with darinaparsin at a concentration starting at 3 μM, which is approximately equal to its IC_50_ value at 24-h post-exposure, in comparison with the similar phenomena observed in the 1 μM-darinaparsin-treated NB4 cells. Intriguingly, almost no necrosis induction was observed in HL-60 cells treated with 3 μM darinaparsin, despite the fact that necrosis, along with apoptosis, was observed in the cells when treated with a relatively high concentration of darinaparsin (5 μM). DNA fragmentation analysis showed further similar results to the annexin V/PI staining results (Figure 2F). These results confirm the higher susceptibility of NB4 cells to darinaparsin when compared with HL-60 cells. The following experiments were thus conducted by exposing NB4 cells to darinaparsin, in order to explore the mechanisms underlying its cytocidal effect against leukemia cells.

### 2.3. Darinaparsin-Triggered Caspase Activation and Bid Truncation in NB4 Cells

**Figure 3:**
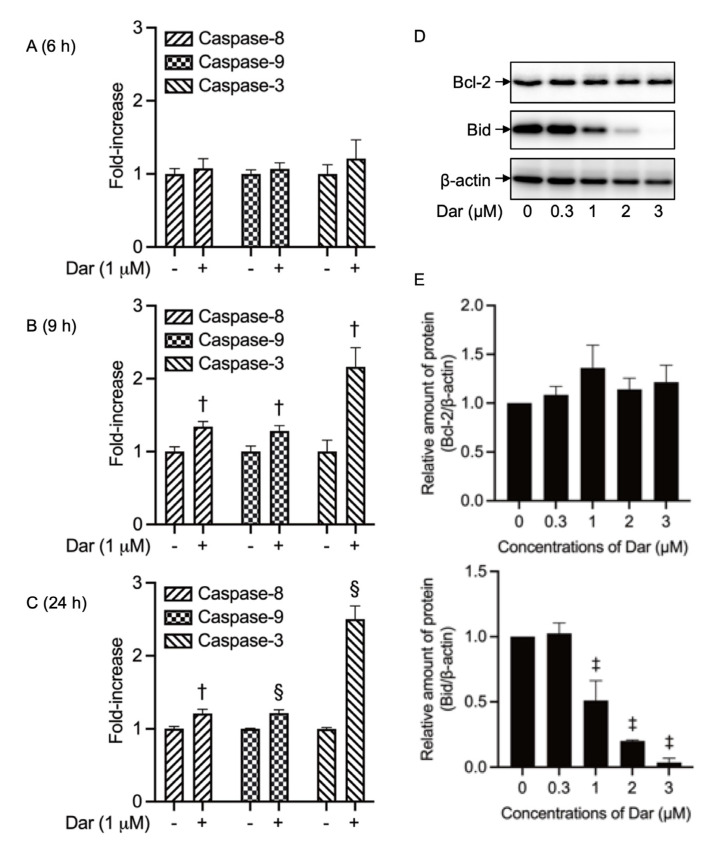
Caspase activation associated with Bid truncation in NB4 cells treated with darinaparsin. Following treatment with 1 µM darinaparsin for 6 h (**A**), 9 h (**B**) and 24 h (**C**). The activities of caspase-8, -9, and -3 were measured using a caspase fluorometric assay kit as described in the “Materials and Methods” section. After treatment with various concentrations of darinaparsin (0.3, 1, 2 and 3 µM) for 24 h, the expression profiles of Bcl-2 and Bid were analyzed using Western blotting. Representative image of the expression profiles of the Bcl-2 and Bid proteins are shown from three independent experiments (**D**). The densitometry of protein bands was analyzed using a program, NIH ImageJ 1.53k. The relative expression levels were expressed as the ratios between each protein and β-actin protein expression levels and were compared with those of an untreated control group (**E**). Results are shown as the means ± SD from three independent experiments. †, *p* < 0.01; §, *p* < 0.001; ‡, *p* < 0.0001 vs. control. Dar, darinaparsin.

Given the critical role of caspases in apoptosis induction, the activities of caspase-8, -9, and -3 were evaluated in NB4 cells after treatment with 1 μM darinaparsin for 6, 9 and 24 h. As shown in Figure 3A–C, although a clear increase in the activity of caspase-8, -9 and -3 was not observed at 6-h post-exposure, the activity of caspase-8 and -9 showed a significant increase at 9-h post-exposure. As expected, the activity of caspase-3, a known downstream target of caspase-8 and -9, was more efficiently intensified by darinaparsin in comparison with that of caspase-8 and -9. The enhanced caspases activation further continued up to 24 h. In addition, as shown in Figure 3D,E, almost no alteration in the expression level of Bcl-2, an anti-apoptotic member of the Bcl-2 family, was observed, which was in agreement with a previous finding showing that a change in Bcl-2 protein expression does not appear to be an obligate feature of the action of darinaparsin [33]. Intriguingly, a dramatic decrease in the expression level of Bid was observed in a dose-dependent manner, indicating that the activation of caspases because of exposure to darinaparsin was accompanied by the cleavage of Bid in NB4 cells.

### 2.4. Suppression of Darinaparsin-Mediated Cell Death by Boc-D-FMK in NB4 Cells

**Figure 4:**
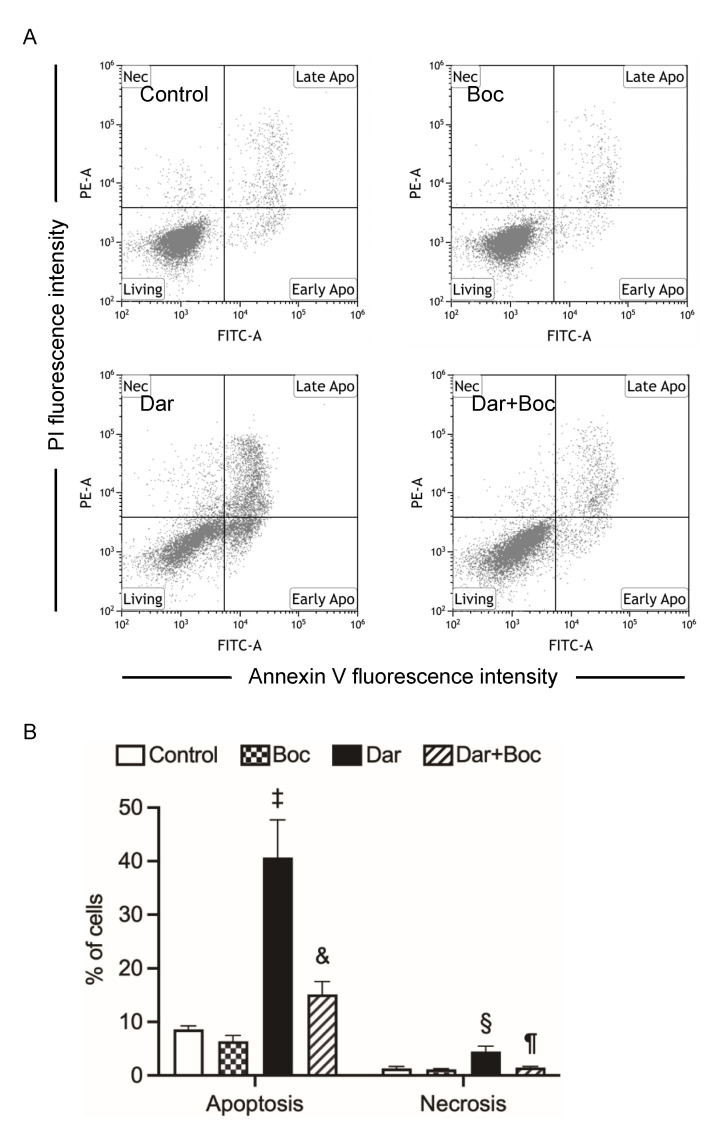
Suppression of darinaparsin-mediated cell death by Boc-D-FMK in NB4 cells. After treatment for 24 h with 1 μM darinaparsin in the presence or absence of 10 μM Boc-D-FMK, a pancaspase inhibitor, cells were stained with annexin V-FITC and PI and analyzed by flow cytometry as described in the legend of Figure 2. Representative flow cytometry dot plots from three independent experiments (**A**), and the quantification in the percentages of apoptotic and necrotic cells (**B**) are shown. Results are shown as the means ± SD from more than three independent experiments. §, *p* < 0.001; ‡, *p* < 0.0001 vs. control; ¶, *p* < 0.01; &, *p* < 0.001 vs. darinaparsin. Dar, darinaparsin; Boc, Boc-D-FMK.

To obtain more detailed information regarding apoptosis induction, the impact of Boc-D-FMK, a pancaspase inhibitor, on darinaparsin-mediated apoptosis was further investigated. Again, exposure of 1 μM darinaparsin for 24 h significantly induced apoptosis along with necrosis in NB4 cells (Figure 4). As expected, the addition of Boc-D-FMK remarkably suppressed the apoptosis induction. Intriguingly, a modest but significant decrease in the proportion of necrotic cells was also observed following the addition of Boc-D-FMK. It is noteworthy that necrotic cells seen in cultures may represent cells that are affected by apoptotic secondary necrosis [34]. The decrease in the proportion of necrotic cells may thus be explained by the ability of Boc-D-FMK to directly inhibit caspase activity and to ultimately suppress the progression of apoptotic secondary necrosis, although further investigation is required to confirm this. In addition, neither apoptosis nor necrosis was influenced by Boc-D-FMK itself.

### 2.5. Suppression of Darinaparsin-Mediated Cell Death by Trolox in NB4 Cells

**Figure 5:**
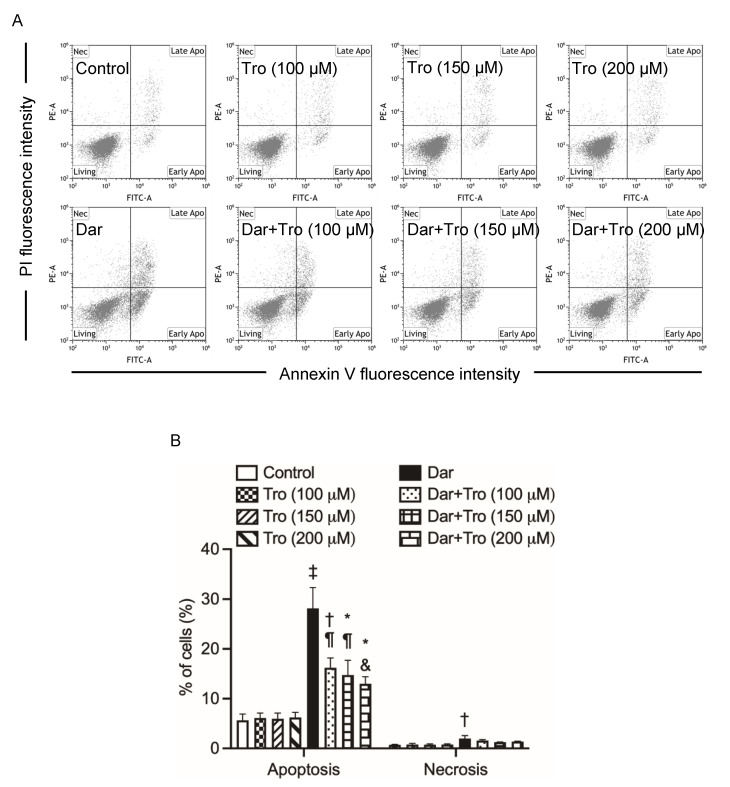
Suppression of darinaparsin-mediated cell death by Trolox in NB4 cells. After treatment for 24 h with 1 μM darinaparsin in the presence or absence of 100, 150 or 200 μM Trolox, an antioxidative reagent, cells were stained with annexin V-FITC and PI and analyzed by flow cytometry as described in the legend of Figure 2. Representative flow cytometry dot plots from three independent experiments (**A**), and the quantification in the percentages of apoptotic and necrotic cells (**B**) are shown. Results are shown as the means ± SD from more than three independent experiments. *, *p* < 0.05; †, *p* < 0.01; ‡, *p* < 0.0001 vs. control; ¶, *p* < 0.01; &, *p* < 0.001 vs. darinaparsin. Dar, darinaparsin; Tro, Trolox.

Previous studies have demonstrated the involvement of oxidative stress in the mechanism of action of darinaparsin [12,33]. To clarify the correlation of oxidative stress with darinaparsin-mediated cell death, NB4 cells were treated with 1 μM darinaparsin for 24 h in the absence or presence of various concentrations of Trolox (100, 150, 200 μM), a vitamin E analogue. As shown in Figure 5, the addition of Trolox significantly suppressed darinaparsin-mediated apoptosis in a dose-dependent fashion. Additionally, a trend towards a decrease in the proportion of necrotic cells was observed. Moreover, it was confirmed that neither apoptosis nor necrosis was altered by Trolox alone.

### 2.6. Effect of Darinaparsin on the Cell Cycle Profiling and the Expression Level of Cell Cycle Related-Gene Proteins in NB4 Cells

**Figure 6:**
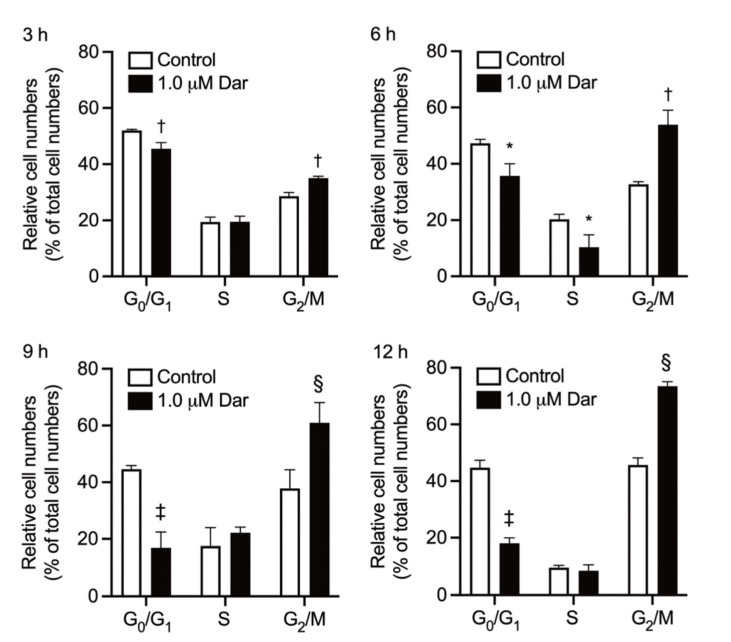
Effect of darinaparsin on cell cycle profiling in NB4 cells. Following treatment with 1 µM darinaparsin for 3, 6, 9 and 12 h, cell cycle analysis was performed by CytoFLEX S flow cytometer as described in the “Materials and Methods” section. Quantifications of the percentages of cells at the G_0_/G_1_, S and G_2_/M phase fractions are shown. Results are shown as the means ± SD from three independent experiments. *, *p* < 0.05; †, *p* < 0.01; §, *p* < 0.001; ‡, *p* < 0.0001 vs. control. Dar, darinaparsin.

Following exposure of NB4 cells to 1 μM darinaparsin for 3, 6, 9, and 12 h, cell cycle analyses were conducted using flow cytometry to evaluate whether cell cycle arrest is involved in the mechanism of action of darinaparsin. As shown in Figure 6 and Supplementary Figure S1, an increase in the number of cells in the G_2_/M phase was identified within 3 h following the treatment and was further enhanced at 6-h post exposure in comparison with control group. Concomitantly, a significant decrease in the number of cells in G_0_/G_1_ and S phase was also observed with time. The exposure of darinaparsin continued to intensify the G_2_/M arrest along with the decrease in the number of cells in the G_0_/G_1_ phase with time, and its cell cycle arrest-inducing activity lasted for at least 12 h. Interestingly, little alteration in the number of cells in the S phase was observed at 9-h and 12-h post-exposure, which might be explained by the dramatic decrease in the number of cells in the G_0_/G_1_ phase, leading to further enhancement in the number of cells in the G_2_/M as well as the increase in the number of cells in the S phase to some degree.

**Figure 7:**
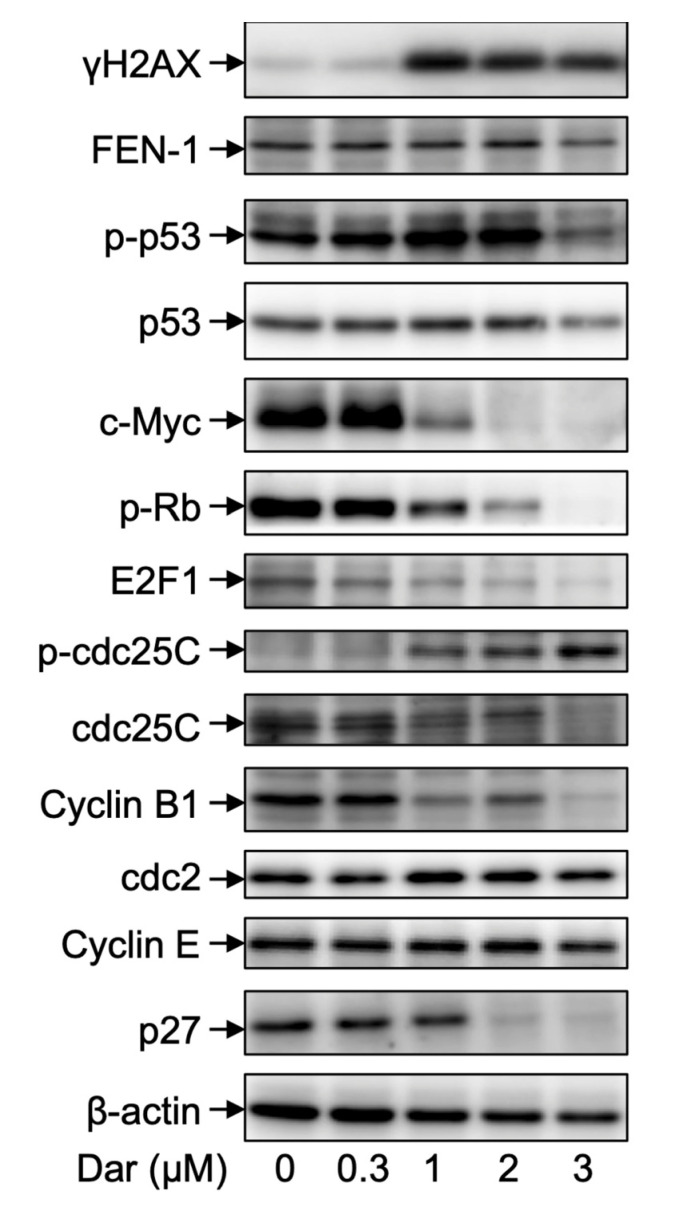
Expression profile of cell cycle arrest-related proteins in NB4 cells treated with darinaparsin. Following treatment with various concentrations of darinaparsin (0.3, 1, 2 and 3 µM) for 24 h, the expression profiles of cell cycle arrest-related proteins were analyzed using Western blotting. Representative image of the expression profile of each key gene protein is shown from three independent experiments. Dar, darinaparsin; p-p53, phosphorylated p53; p-Rb, phosphorylated Rb; p-cdc25C, phosphorylated cdc25C. The images of beta-actin are identical to those in Figure 3D because the same experiment samples were used for analysis.

**Figure 8:**
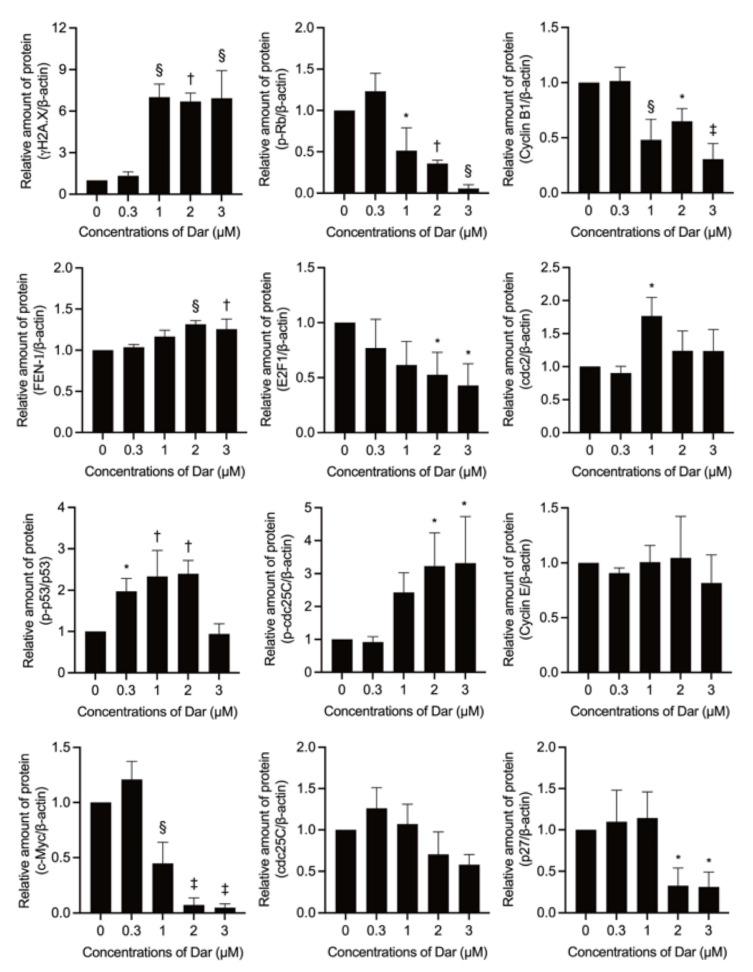
Alteration of expression level of cell cycle arrest-related proteins in NB4 cells treated with darinaparsin. Following treatment with various concentrations of darinaparsin (0.3, 1, 2 and 3 µM) for 24 h, the alteration of expression levels of cell cycle arrest-related proteins was analyzed according to the Western blot images shown in Figure 7. The densitometry of protein bands was analyzed using a program, NIH ImageJ 1.53k. The relative expression levels were expressed as the ratios between each key gene protein and β-actin protein expression levels and compared with those of the untreated control group. Results are shown as the means ± SD from three independent experiments. *, *p* < 0.05; †, *p* < 0.01; §, *p* < 0.001; ‡, *p* < 0.0001 vs. control. Dar, darinaparsin; p-p53, phosphorylated p53; p-Rb, phosphorylated Rb; p-cdc25C, phosphorylated cdc25C.

Given a close correlation between cell cycle arrest and chemotherapeutic drug-mediated DNA damage, the expression of γH2AX, a DNA damage marker [35,36], was first evaluated in NB4 cells following exposure to 0.3, 1, 2, and 3 μM darinaparsin for 24 h. As shown in Figure 7 and Figure 8, the expression level of γH2AX was dramatically upregulated in the cells treated with darinaparsin at a concentration equal to or greater than 1 μM, although there was only a slight increase in its expression when treated with 0.3 μM darinaparsin. By contrast, a small but significant increase in the expression of flap endonuclease 1 (FEN1), known as an important factor in DNA damage repair [35,37], was observed only in the cells treated with relatively high concentrations of darinaparsin (2 and 3 μM). Transcription factors p53 and c-Myc are known to be involved in regulating proliferation and apoptosis induction [20,21,38]. In parallel with the dramatic upregulation of γH2AX, a substantial and significant increase in the expression levels of phosphorylated p53 (p-p53) over the endogenous levels was observed in the cells treated with darinaparsin even at the concentration as low as 0.3 μM, indicating p53 activation. Interestingly, the degree of p53 activation almost returned to control level when treated with a relatively high concentration of 3 μM, indicating that a transient increase in its activation might be required for the action of darinaparsin. Additionally, darinaparsin induced a remarkable decrease in the expression level of c-Myc in a dose-dependent manner. Retinoblastoma (Rb) tumor suppressor protein is known to be regulated by c-Myc and closely related to cell cycle regulation and apoptosis induction [39,40]. In this regard, treatment with darinaparsin caused a dose-dependent reduction in the expression of phosphorylated Rb (p-Rb), the inactive form of Rb, indicating the activation of Rb. The expression level of E2F1, a downstream target of Rb action [39,40], was concomitantly downregulated in a similar manner, indicating the inactivation of E2F1.

An increased level of the inactive phosphorylated form of cdc25C (Ser216) (p-cdc25C), along with a clear decrease in its total form was also observed in darinaparsin-treated NB4 cells. Coincidentally, the expression level of Cyclin B1 was remarkably downregulated following exposure to darinaparsin at a concentration equal to or greater than 1 μM, although only a slight but significant increase in the expression level of cdc2 was observed in 1 μM darinaparsin-treated cells. Of note, the expression level of p27 was dramatically downregulated by 2 μM and 3 μM darinaparsin. In addition, almost no significant alteration in the expression level of Cyclin E was detected, and the expression level of p21 and Cyclin D1 was hardly detected regardless of the treatment of darinaparsin, indicating little correlation between these players and darinaparsin-mediated G_2_/M arrest.

### 2.7. Effect of Trolox on G_2_/M Phase Arrest in NB4 Cells Treated with Darinaparsin

**Figure 9:**
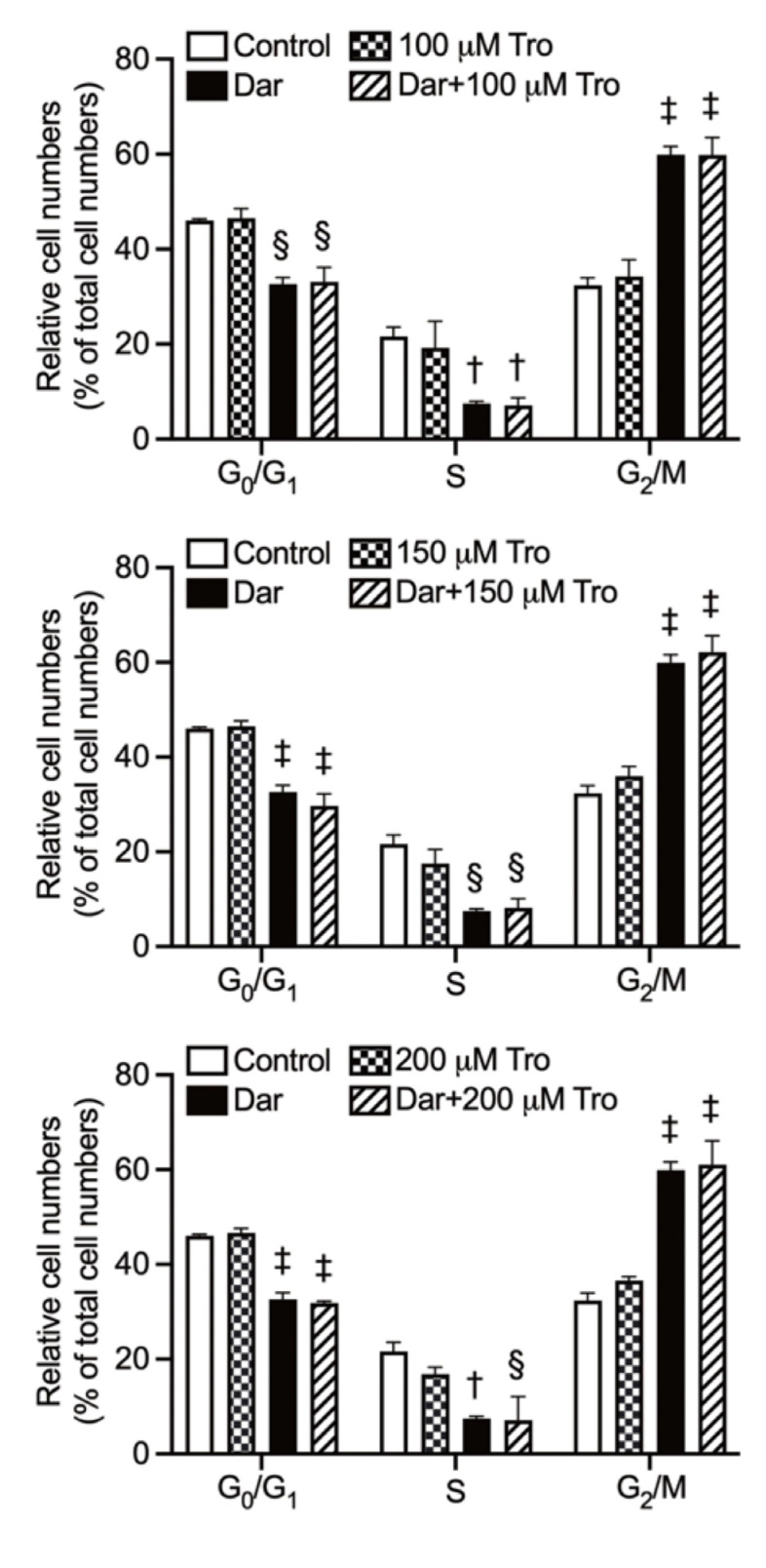
Effect of Trolox on cell cycle profiling in NB4 cells treated with darinaparsin. After treatment for 6 h with 1 μM darinaparsin in the presence or absence of 100, 150 and 200 μM Trolox, cell cycle analysis was performed as described in the legend of Figure 6. Quantification of the percentages of cells at the G_0_/G_1_, S and G_2_/M phase fractions are shown. Results are shown as the means ± SD from more than three independent experiments. †, *p* < 0.01; §, *p* < 0.001; ‡, *p* < 0.0001 vs. control. Dar, darinaparsin; Tro, Trolox.

Consistent with results in Figure 6, exposure to NB4 cells with 1 μM darinaparsin for 6 h induced G_2_/M arrest along with a significant decrease of the number in G_0_/G_1_ and S phase cells (Figure 9 and Supplementary Figure S2). As shown in Figure 9, almost no alteration in the cell cycle profiling of darinaparsin-treated NB4 cells was observed, regardless of the addition of 100, 150 or 200 μM Trolox. In addition, it was confirmed that the cell cycle profiling was not altered by Trolox alone.

### 2.8. Effect of Darinaparsin on Notch1 Signaling-Related Gene Protein Expression in NB4 Cells

**Figure 10:**
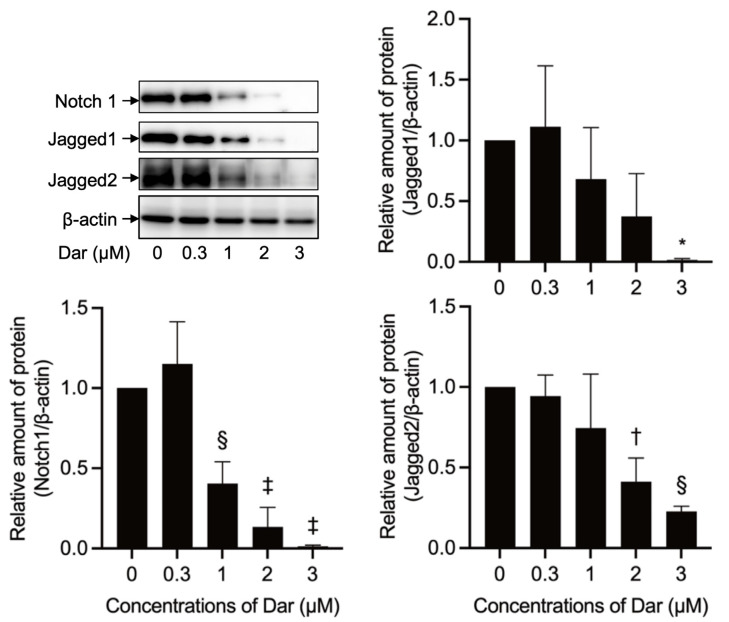
Effect of darinaparsin on Notch1 signaling-related gene protein expression. Following treatment with various concentrations of darinaparsin (0.3, 1, 2 and 3 µM) for 24 h, the alteration of expression levels of Notch1 signaling-related proteins was analyzed using Western blot. Representative images of the expression profiles of each protein are shown from three independent experiments. The relative expression levels are expressed as the ratios between each gene protein and β-actin protein expression levels and are compared with those of untreated control group. Results are shown as the means ± SD from three independent experiments. *, *p* < 0.05; †, *p* < 0.01; §, *p* < 0.001; ‡, *p* < 0.0001 vs. control. Dar, darinaparsin. The images of beta-actin are identical to those in Figure 3D because the same experiment samples were used for analysis.

Deregulated Notch1 signaling has been implicated in various types of cancer including T-cell lymphoblastic leukemia/lymphoma [29,30]. As shown in Figure 10, the expression of Notch1 and its ligand Jagged1 and Jagged2 proteins was successfully detected in control groups. Of note, the exposure of NB4 cells to darinaparsin caused a significant, dose-dependent downregulation of the expression levels of Notch1, Jagged1 and Jagged2.

## 3. Discussion

Results from this study clearly demonstrate that darinaparsin was more potently cytotoxic than As^III^ to all four leukemia cell lines tested (Figure 1). Similarly, darinaparsin has been shown to induce a dose-dependent apoptosis in a As_2_O_3_-resistant myeloma cell line [13]. In addition, Bansal et al. have demonstrated that darinaparsin may serve as an inhibitor of Hedgehog signaling to inhibit prostate tumor-initiating cells and Du145 prostate tumor xenografts [11]. A series of clinical studies have further demonstrated the antitumor activity and favorable safety in patients with relapsed/refractory lymphoma including PTCL [14,15,41]. Overall, these findings suggest the great potential clinical application of darinaparsin in patients with different types of cancer. In fact, a previous study monitoring the response and resistance to darinaparsin in an AML patient with inversion of chromosome 3 (inv(3)(q21q26.2)) has suggested that darinaparsin may be a good treatment option for inv(3) AML [42].

In line with previous reports [13,26], we have further demonstrated that darinaparsin induced apoptosis in both NB4 and HL-60 cells in a dose-dependent manner (Figure 2). Notably, necrosis was also observed in NB4, but not HL-60 cells, following the exposure to darinaparsin at a concentration equal to their respective IC_50_ values, reconfirming the higher susceptibility of NB4 cells. Additionally, treatment with a relatively high concentration of darinaparsin (5 μM) caused both apoptosis and necrosis in HL-60 cells (Figure 2D,E). Considering that the induction of necrotic cell death in chemotherapeutic treatments has become increasingly appreciated due to the ability of tumor cells to evolve diverse strategies to evade apoptosis during tumor development [43], the capability of darinaparsin to induce both apoptosis and necrosis provides a competitive advantage in enhancing its antitumor activity and overcoming drug resistance. Our results also demonstrate that darinaparsin triggered the activation of caspase-8, -9, and -3, and that the enhanced caspases activation continued up to 24 h (Figure 3A). A remarkable suppression of apoptosis induction by Boc-D-FMK also provided evidence in support of the critical role of caspases in darinaparsin-triggered apoptosis (Figure 4). In comparison to the absence of alteration in Bcl-2 expression, a substantial decrease in the expression level of Bid was observed in a dose-dependent manner (Figure 3D,E). Bid is a key molecule in the regulation of apoptosis, and caspase-8-mediated cleavage of Bid into its pro-apoptotic truncated active form reveals a direct connection between the intrinsic and extrinsic pathway [17,19]. Taken together, to the best of our knowledge, our results suggest, for the first time, that darinaparsin triggered a convergence of the intrinsic and extrinsic pathways of apoptosis via the activation of caspase-8 and cleaved Bid; though a previous study has previously suggested that treatment with darinaparsin results in activation of both extrinsic and intrinsic pathways in two T-cell lymphoma cell lines, Jurkat and Hut78 [26]. The suppression of darinaparsin-mediated apoptosis by Trolox in the current study (Figure 5) provides further evidence to support previous opinions regarding the involvement of oxidative stress in the mechanism of action of darinaparsin [12,27,33]. Diaz et al. have demonstrated that darinaparsin is more potent than As_2_O_3_ at inducing oxidative damage in NB4 cells and their arsenic-resistant subclone AR2 cells [27], supporting the suggestion of the greater potency of darinaparsin against all leukemia cells tested in the current study. Of note, the addition of Trolox, ranging from 100 to 200 μM, provided only partial inhibition of apoptosis and had a limited effect on necrosis (Figure 5). It has been demonstrated that exposure to heavy metals, including arsenic, caused various types of DNA damage due to their capability to directly induce double-strand breaks (DSBs) as well as reactive oxygen species [24,25]. Taking these previous results, and our observations into account, we suggest that darinaparsin-mediated apoptotic/necrotic cell death can be partially attributed to forms of DNA damage such as DSBs, which might not be completely abolished by Trolox.

We further showed the capability of darinaparsin to induce G_2_/M phase arrest in NB4 cells (Figure 6). Cell cycle arrest-inducing activity has been implicated in the antitumor activity of darinaparsin in cancer cell lines derived from different types of tumors including lymphoma and prostate carcinoma [11,26]. Additionally, Bansal et al. have demonstrated that darinaparsin induces G_2_/M phase arrest, but not apoptosis in a prostate cancer cell line Du145 [11]. Darinaparsin has also been demonstrated to radiosensitize cancer cells but, interestingly, to protect normal intestinal epithelial cells from radiation; a phenomenon that darinaparsin-mediated G_1_/S and G_2_/M cell cycle-arrest contributes to by enhancing DNA damage repair [44]. Collectively, these findings suggest that the generality of the mechanism underlying the effects of darinaparsin on normal or cancerous cells is largely attributed to its ability to modulate cell cycle arrest, although further investigation is obviously needed.

In agreement with previous reports showing that chemotherapeutic agents could influence the competitive balance between DNA damage and its repair mechanisms and ultimately exhibit antitumor activity [25], a dramatic upregulation of γH2AX, a DNA damage marker [35,36], was observed in darinaparsin-treated NB4 cells (Figure 7 and Figure 8). By contrast, only a small increase in the expression of FEN1, a factor in DNA damage repair [35,37], was confirmed, suggesting the compensatory increase in response to the DNA damage induced by darinaparsin. DNA damage can halt cell cycle progression by upregulating p53 and/or downregulating c-Myc and can subsequently result in G_2_/M arrest as well as apoptosis [20,21,22,23,38]. The abnormal expression of cdc25C has been linked to disease progression and poor prognosis in many cancer types, and its downregulation could induce cell cycle arrest in the G_2_/M phase in response to DNA damage via p53-mediated signal transduction [45]. Cdc25C has been found to be responsible for promoting and maintaining the activation of cyclin B1/cdc2, and to ultimately determine G_2_/M progression [45,46]. In addition, downregulation of c-Myc has been shown to modulate the Rb/E2F pathway, leading to G_2_/M arrest [21]. It has been shown that non-phosphorylated Rb, the active form of Rb, can bind E2F1 and consequently result in the inactivation of E2F1 and inhibition of the cell cycle [21,23,47]. In the current study, the activation of p53 and downregulation of c-Myc were first observed in NB4 cells treated by darinaparsin. In parallel with the activation of p53, inhibition of cdc25C, evidenced by the increase of its inactive form p-cdc25C and a clear decrease in its total form, together with the downregulation of Cyclin B1 were confirmed, though a modest increase in the expression level of cdc2 was coincidentally detected (Figure 7 and Figure 8), suggesting that Cyclin B1 was primarily targeted by p-53/cdc25C instead of cdc2. As expected, the activation of Rb and inactivation of E2F1 occurred in parallel with the downregulation of c-Myc, suggesting that c-Myc contributed to darinaparsin-triggered G_2_/M arrest by modulating Rb/E2F1 pathway.

Surprisingly, a dramatic downregulation of p27 was induced by a relatively high concentration of darinaparsin (2 and 3 μM) (Figure 7 and Figure 8). It should be noted that p27 protein levels are elevated in CML leukemic blasts [48]. Wegiel et al. report that a statistically significant correlation between the expression of vascular endothelial growth factor (VEGF) and of p27 was observed in bone marrows from 42 patients with AML, and further demonstrated that increased p27 expression enhances the ability of VEGF and VEGFR-2 to promote the migration of U-937 cells, suggesting a role for p27 in cell migration that might be independent of its role in the cell cycle [49]. Collectively, the inhibition of p27 observed in the current study might be linked to the ability of darinaparsin to suppress cancer cell migration/invasion. It also suggests that the correlation between p27 downregulation by darinaparsin and migration/invasion warrants further investigation. Additionally, Trolox successfully suppressed apoptosis induction, though it failed to correct G_2_/M phase arrest in NB4 treated with darinaparsin (Figure 9), suggesting that darinaparsin-triggered oxidative stress primarily contributed to apoptosis induction rather than to G_2_/M phase arrest.

Aberrant activation in the Notch1 signaling pathway has been demonstrated to be implicated in hematological malignancies including T-cell lymphoblastic leukemia/lymphoma [28,29,30]. In the current study, suppression of Notch1 signaling was confirmed as evidenced by downregulation of the expression levels of Notch1 and its ligands Jagged1/Jagged2 in NB4 cells treated with darinaparsin (Figure 10). In addition, As_2_O_3_ has been shown to inhibit cell proliferation and induce apoptosis in malignant cells by suppressing Notch1 signaling [31,32]. Collectively, these results suggest that inactivation of Notch1 signaling probably contributes to the cytotoxic effect of darinaparsin in NB4 cells.

## 4. Materials and Methods

### 4.1. Materials

Darinaparsin (Lot. FP-000734) was provided by Solasia Pharma K.K., (Tokyo, Japan). Sodium arsenite (NaAsO_2_, As^III^) was purchased from Tri Chemical Laboratories (Yamanashi, Japan). Fetal bovine serum (FBS) was purchased from Sigma Chemical Co. (St. Louis, MO, USA) and Nichirei Biosciences (Tokyo, Japan). RPMI-1640 (without phenol red), protease inhibitor mixture (×100), penicillin-streptomycin solution (×100), dimethyl sulfoxide (DMSO) and ClearTrans^®^ SP PVDF membrane were obtained from Wako Pure Chemical Industries (Osaka, Japan). Cellstain^®^ Hoechst 33342 solution, 2-(4-Iodophenyl)-3-(4-nitrophenyl)-5-(2,4-disulfophenyl)-2*H*-tetrazolium (WST-1), 1-methoxy-5-methylphenazinium methylsulfate (1-Methoxy PMS) and 2-(4-(2-Hydroxyethyl)-1-piperazinyl) ethanesulfonic acid (HEPES) were obtained from Dojindo Molecular Technologies, Inc. (Tokyo, Japan). Trolox (6-hydroxyl-2,5,7,8-tetramethylchroman-2-carboxylic acid, a water-soluble analog of Vitamin E) and BOC-Asp(OMe)-fluoromethyl ketone (Boc-D-FMK), a pancaspase inhibitor, were purchased from Tokyo Chemical Industry (Tokyo, Japan), and ChemScene LLC (Monmouth Junction, NJ, USA), respectively. The stock solution of 100 mM Boc-D-FMK was prepared by dissolving the compound in DMSO and storing it at −20 °C for future use. Can Get Signal^®^ Immunoreaction Enhancer Solution was purchased from Toyobo CO., LTD. (Osaka, Japan). Bovine Serum Albumin (BSA) standard and Quick Start™ Bradford 1 × Dye Reagent were purchased from Thermo Fisher Scientific (Rockford, IL, USA) and BIO-RAD (Berkeley, CA, USA), respectively.

### 4.2. Cell Culture and Treatment

NB4, a human APL cell line with t(15;17), was obtained from the Deutsche Sammalung von Mikroorganismen und Zellkulturen GmbH (Braunschweig, Germany). A human promonocytic leukemia cell line, U-937, and a human acute lymphoblastoid leukemia cell line, MOLT-4, were obtained from the American Type Culture Collection (ATCC, Manassas, VA, USA). A human myeloid leukemia cell line, HL-60, was obtained from the RIKEN cell bank (Ibaraki, Japan). All cell lines were cultured in RPMI-1640 medium supplemented with 10% heat-inactivated FBS (Sigma for NB4 and HL-60; Nichirei for U-937 and MOLT-4), 100 U/mL of penicillin and 100 µg/mL of streptomycin in a humidified 5% CO_2_ atmosphere at 37 °C.

### 4.3. Cell Viability Assay

The cytotoxicity of darinaparsin was measured by the WST-1 assay as described previously [50]. Briefly, 5.5 mM WST-1 and 2 mM 1-Methoxy PMS were prepared by dissolving each regent in 20 mM HEPES (pH 7.4). The cells were seeded in 96-well plates (SUMILON, Tokyo, Japan) at a density of 2 × 10^5^ cells/well in 0.2 mL medium, followed by treatment for 24 h with various concentrations of darinaparsin. Then, 15 μL of the mixture of WST-1 and 1-Methoxy PMS (9:1 (*v*/*v*)) were added into each well. After incubation at 37 °C for 1 h, the plates were mixed and the absorbance at 450 nm was measured with a microplate reader (SpectraMax M5^e^, Molecular Devices, San Jose, CA, USA). The relative cell viability was expressed as the ratio of the absorbance of each treatment group against those of the corresponding untreated control group. Data are shown as means ± standard deviation (SD) from more than three independent experiments. The IC_50_ values of darinaparsin against each cell line were calculated using GraphPad Prism^®^9 software.

### 4.4. Determination of Apoptosis

#### 4.4.1. Annexin V/PI Analysis

The Annexin V FITC apoptosis detection Kit (Cayman Chemical, Ann Arbor, MI, USA) was used for the detection of apoptotic and necrotic cells according to the method described previously [19,51]. Briefly, following the treatment for 24 h with indicated concentrations of darinaparsin, cells were washed with PBS. Cells were then incubated for 10 min in 50 μL of reaction buffer, which containing annexin V-FITC and propidium Iodide (PI) from the kit, followed by the addition of 150 μL of binding buffer. Fluorescence intensities of FITC and PI were measured by a CytoFLEX flow cytometer (Beckman Coulter, Brea, CA, USA). A total of 10,000 events were acquired and data were analyzed by CytExpert Ver 2.4.0.28 software (Beckman Coulter, CA, USA). In experiments using Boc-D-FMK or Trolox, cells were preincubated with the respective compound at the indicated concentrations for 1 h. Since DMSO was used to prepare the stock solution of Boc-D-FMK, control samples were prepared by treating cells with culture medium containing solely the vehicle reagent, DMSO (final concentration: 0.1%).

#### 4.4.2. DNA Gel Electrophoresis

Preparation and agarose gel electrophoresis of DNA were carried out according to the methods reported previously [19]. Extracted DNA was dissolved in TE buffer (10 mM Tris–HCl, pH 8.0, 1 mM EDTA), and the DNA concentration was determined by measuring absorbance at 260 nm on a BioSpec-nano spectrophotometer (Shimadzu, Tokyo, Japan). The DNA samples (20 μg in 20 μL) and a Read-Load™100 bp DNA Ladder (Nippon gene, Tokyo, Japan) as a DNA size marker were electrophoresed on a 2% agarose gel (Agarose X, Wako Pure Chemical Industries, Osaka, Japan) using TBE buffer (89 mM Tris, 89 mM boric acid, 2 mM EDTA). Gels were stained with ethidium bromide (Sigma Ltd. St. Louis, MO, USA) and then viewed under CCD FUNA-BOX2 Gel Studio (Funakoshi Co., Tokyo, Japan).

### 4.5. Measurement of Caspases Activity

Activities of caspase-9, -8, and -3 were measured using the caspase fluorometric assay kit according to the method described previously [51,52]. Briefly, protein samples (25 μg/50 μL) were plated on a 96-well black plate, followed by the addition of 50 μL of 2 × reaction buffer containing 10 mM DTT to each sample, and then 5 μL of 1 mM caspase substrate (final concentration of 50 μM). After incubation at 37 °C for 1 h, the fluorescent intensity (Excitation. 400 nm, Emission. 505 nm) was measured using a microplate reader (SpectraMax Pro M5^e^, Molecular Devices, USA).

### 4.6. Cell Cycle Analysis

After treatment with 1 µM darinaparsin in the presence or absence of Trolox at the concentrations indicated, cell cycle analysis was performed using a CytoFLEX S flow cytometer (Beckman Coulter, CA, USA) according to the methods previously described with slight modifications [52,53]. Briefly, cells were washed twice with cold PBS, fixed with 1% paraformaldehyde/PBS on ice for 30 min, washed twice again with cold PBS, permeabilized in 70% (*v*/*v*) cold ethanol and kept at −20 °C for at least 4 h. Cell pellets were then washed twice with cold PBS after centrifugation (430× *g* for 5 min at 4 °C), and resuspended in 500 µL of Hoechst 33342/PBS (4 µg/mL of Hoechst 33342 in PBS), followed by incubation for 30 min in the dark at room temperature. A total of 10,000 events were acquired for flow cytometric analysis using CytExpert Ver 2.4.0.28 software. Kaluza Analysis 2.1 software (Beckman Coulter, CA, USA) was used to calculate the number of cells at the G_0_/G_1_, S and G_2_/M phase fractions.

### 4.7. Western Blot Analysis

For preparation of the protein samples, cell pellets (approximately 1–2 × 10^6^ cells per 110 μL buffer) were suspended in Laemmli buffer containing protease inhibitor mixture. Cell suspensions were sonicated (Qsonica, LLC, Newtown, CT, USA) with 10 short bursts of 2 s followed by intervals of 2 s for cooling. The suspensions were kept in an ice bath. Sonicated cells were heated in 95 °C for 5 min, and then centrifuged at 13,000× *g* for 15 min at 4 °C. Protein concentrations of the supernatant were determined according to Bradford’s method using the protein dye reagent, according to the manufacturer’s instructions, and using BSA as the standard. Western blot analysis was carried out according to the methods previously described [53,54]. Briefly, after separation of proteins (10–20 μg protein/lane) on a sodium dodecyl sulfate (SDS) polyacrylamide gel electrophoresis, followed by their transference to a PVDF membrane, protein bands were detected using the following primary antibodies and dilution ratios: rabbit anti-human β-actin (1:1000 dilution; cat. no. 4967), rabbit anti-human Bid (1:1000 dilution; cat. no. 2002), rabbit anti-human phospho-Histone H2AX (Ser139) (1:1000 dilution; cat. no. 9718), mouse anti-phospho-p53 (Ser15) (16G8) (1:1000 dilution; cat. no. 9286), rabbit anti-human phospho-Rb (1:1000 dilution; cat. no. 9308), rabbit anti-human E2F1 (1:1000 dilution; cat. no. 3742), rabbit anti-human phospho-cdc25C (Ser216) (1:1000 dilution; cat. no. 9528), rabbit anti-human cdc25c (1:1000 dilution; cat. no. 4688), mouse anti-human Cyclin B1 (1:2000 dilution; cat. no. 4135), mouse anti-human cdc2 (1:1000 dilution; cat. no. 9116), rabbit anti-human Cyclin D1 (1:1000 dilution; cat. no. 2978), rabbit anti-human Cyclin E (1:1000 dilution; cat. no. 20808), mouse anti-human p21 (1:2000 dilution; cat. no. 2946), rabbit anti-human p27 (1:1000 dilution; cat. no. 2552), rabbit anti-human Notch1 (1:1000 dilution; cat. no. 4380; all from Cell Signaling Technology, Inc., Danvers, MA, USA); mouse anti-human Bcl-2 (1:1000 dilution; cat. no. B3170; Sigma–Aldrich, Louis, MO, USA); mouse anti-human FEN-1 (1:1000 dilution; cat. no. NB100-150; Novus Biologicals, LLC, Centennial, CO, USA); mouse anti-human p53 (1:1000 dilution; cat. no. sc-126), mouse anti-human c-Myc (1:500 dilution; cat. no. sc-40), mouse anti-human Jagged1 (1:500 dilution; cat. no. sc-390177), mouse anti-human Jagged2 (1:500 dilution; cat. no. sc-515725; all from Santa Cruz, CA, USA). Blotted protein bands were detected with respective horseradish peroxidase-conjugated secondary antibody and an enhanced chemiluminescence (ECL) Western blot analysis system (Amersham Pharmacia Biotech, Buckinghamshire, UK). Relative amounts of the immunoreactive proteins were calculated from the density of the gray level on a digitized image using a program, NIH ImageJ 1.53k.

### 4.8. Statistical Analysis

Experiments were independently repeated three times and reported as the means ± SD of the three assays. Statistical analysis was conducted using one-way ANOVA followed by Dunnett’s post-test. In the experiments using inhibitors, statistical analysis was conducted using one-way ANOVA followed Tukey’s post-test. A probability level of *p* < 0.05 was considered statistically significant.

## 5. Conclusions

Our results demonstrate that darinaparsin showed more potent cytotoxicity than As^III^ against the tested leukemia cell lines and induced apoptosis/necrosis in both NB4 and HL-60 cells. Both NB4 and HL-60 are human acute myeloid leukemia cells, regardless of whether they harbor the t(15;17) translocation leading to the expression of the fusion protein promyelocytic leukemia-retinoic acid receptor α (PML-RARα) [1]. Since darinaparsin showed potent in vitro anticancer properties against both cells, darinaparsin might be successfully deployed in clinical applications for the treatment of patients with different subtypes of AML, although a larger scale randomized study should be launched to draw a solid conclusion. Detailed investigation into the effects of darinaparsin on NB4 cells showed the occurrence of a convergence of the intrinsic and extrinsic pathways of apoptosis via the activation of caspase-8 and cleaved Bid. G_2_/M arrest associated with DNA damage, and the activation of p53 along with the inhibition of cdc25C/cyclin B1/cdc2 were observed in NB4 cells treated with darinaparsin. Downregulation of c-Myc was likely to contribute to the G_2_/M arrest by modulating the Rb/E2F1 pathway. We further suggest that darinaparsin-triggered oxidative stress primarily contributed to apoptosis induction rather than to G_2_/M arrest. Additionally, suppression of Notch1 signaling probably contributed to the cytotoxicity of darinaparsin. Further investigation into the mechanism of action of darinaparsin in terms of the abovementioned molecules in tumor xenografts is warranted.

## Data Availability

All data generated or analyzed during this study are included in this published article.

## Supplementary Materials

The supporting information can be downloaded at: https://www.mdpi.com/article/10.3390/ijms24032282/s1.

## Abbreviations

PTCL: peripheral T–cell lymphoma; AML: acute myeloid leukemia; CML: chronic myeloid leukemia; APL: acute promyelocytic leukemia; As^III^: trivalent arsenic (arsenite); As_2_O_3_: arsenic trioxide; SSBs: single-strand breaks; DSBs: double-strand breaks; FEN1: flap endonuclease 1; Rb: retinoblastoma; VEGF: vascular endothelial growth factor; VEGFR: vascular endothelial growth factor receptor.
