# Supplementary figures and images for: Cytotoxic Effects of Darinaparsin, a Novel Organic Arsenical, against Human Leukemia Cells
Source: Int J Mol Sci. 2023 Jan 23;24(3):2282. doi: 10.3390/ijms24032282 (PMC9916914; doi:10.3390/ijms24032282)

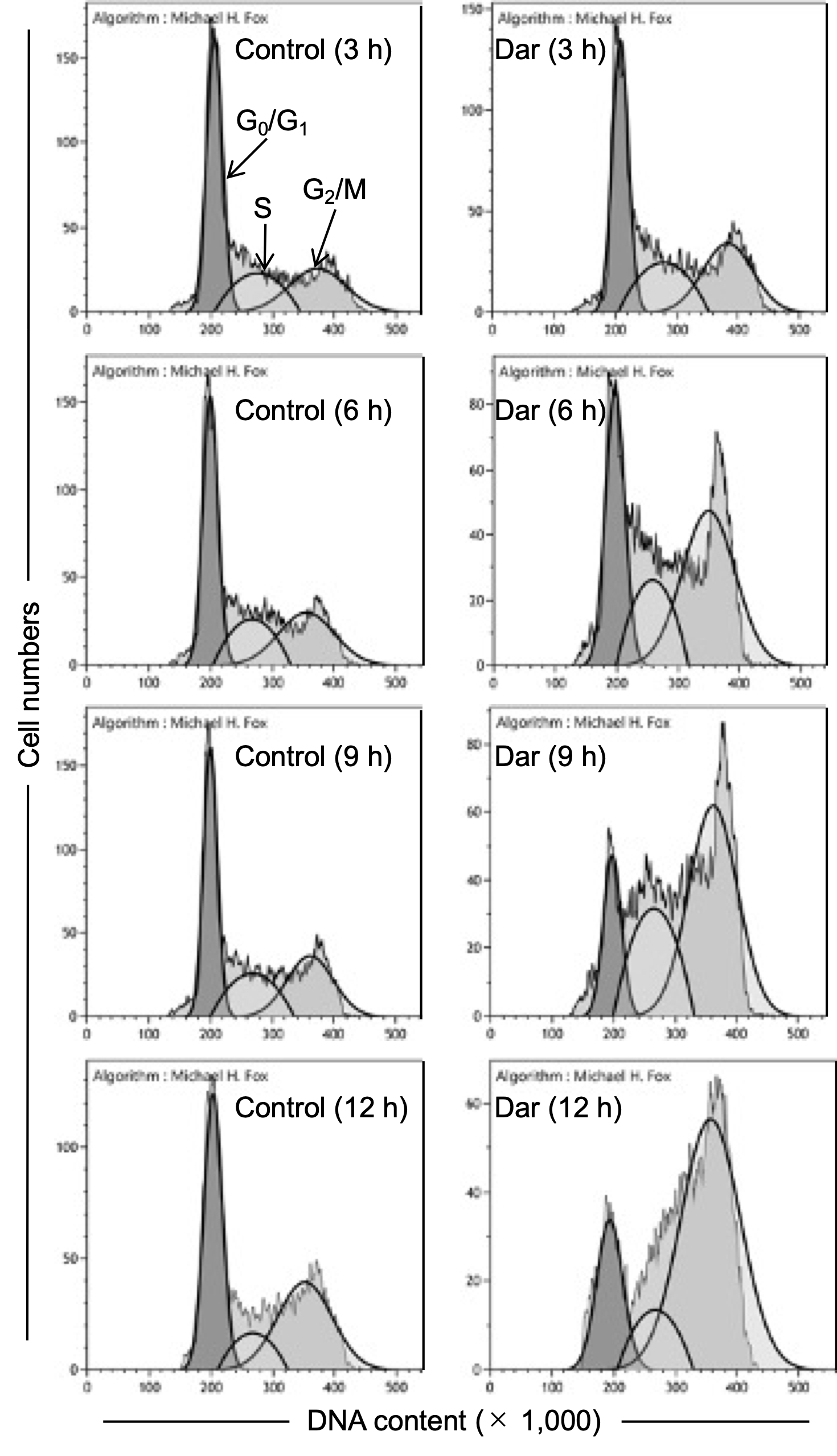

Supplement: Supplementary file 1 [file ijms-24-02282-s001.zip › Figure S1.tiff]

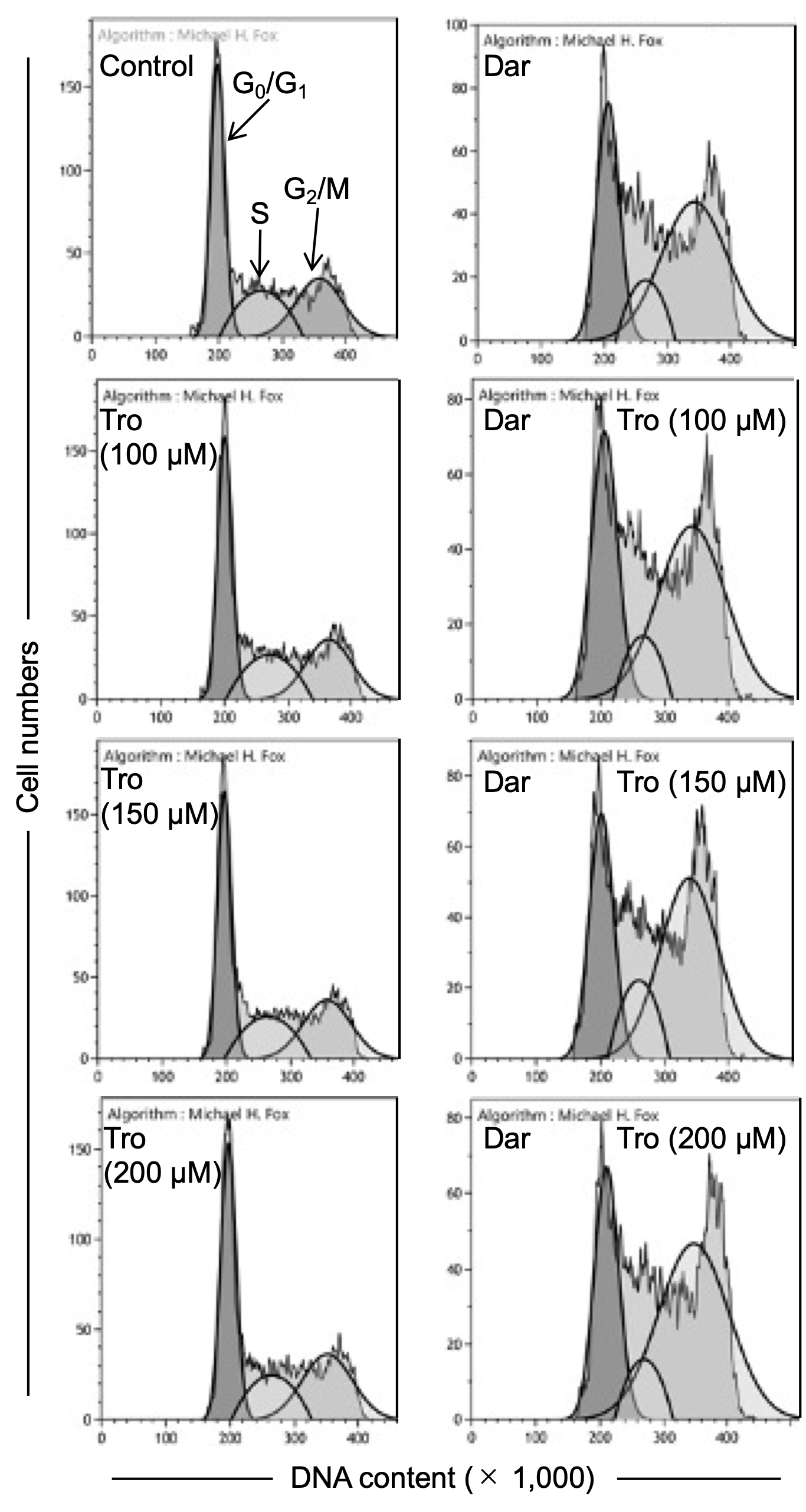

Supplement: Supplementary file 1 [file ijms-24-02282-s001.zip › Figure S2.tiff]

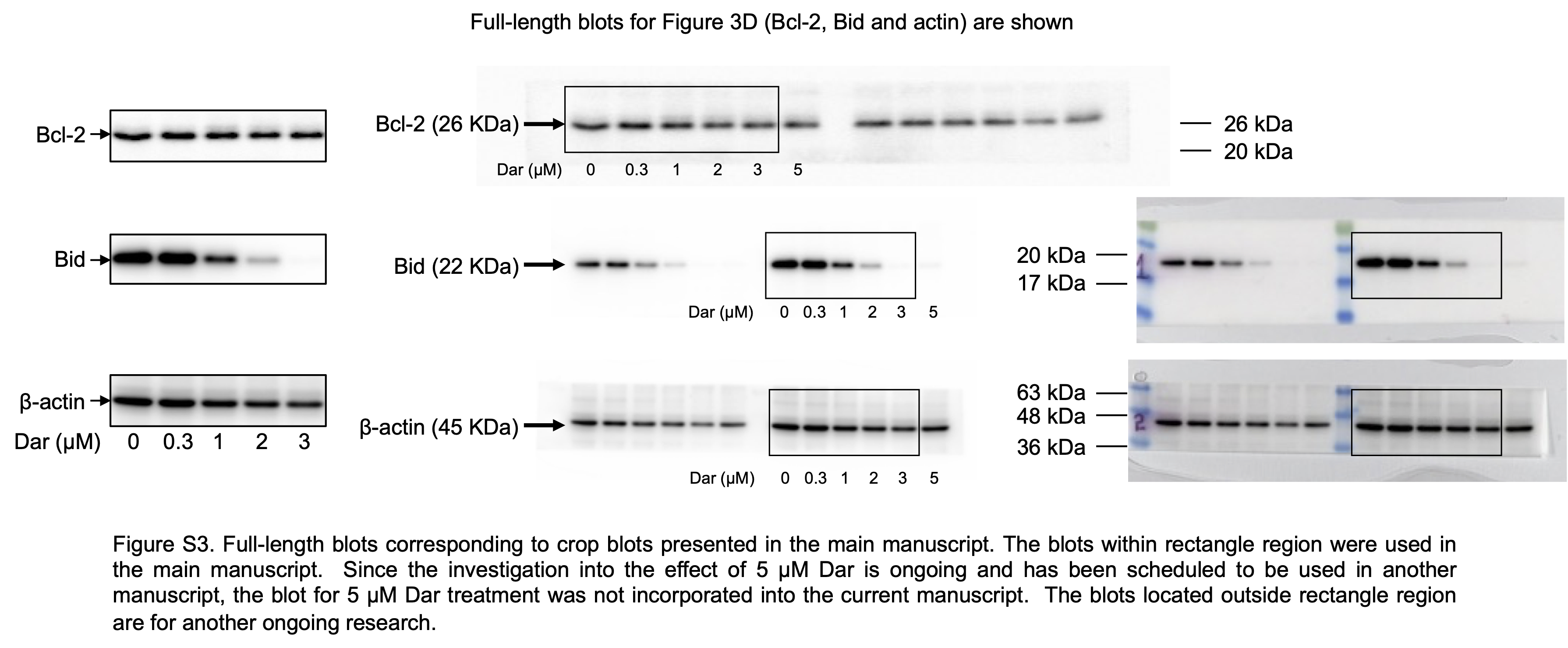

Supplement: Supplementary file 1 [file ijms-24-02282-s001.zip › Figure S3.tiff]

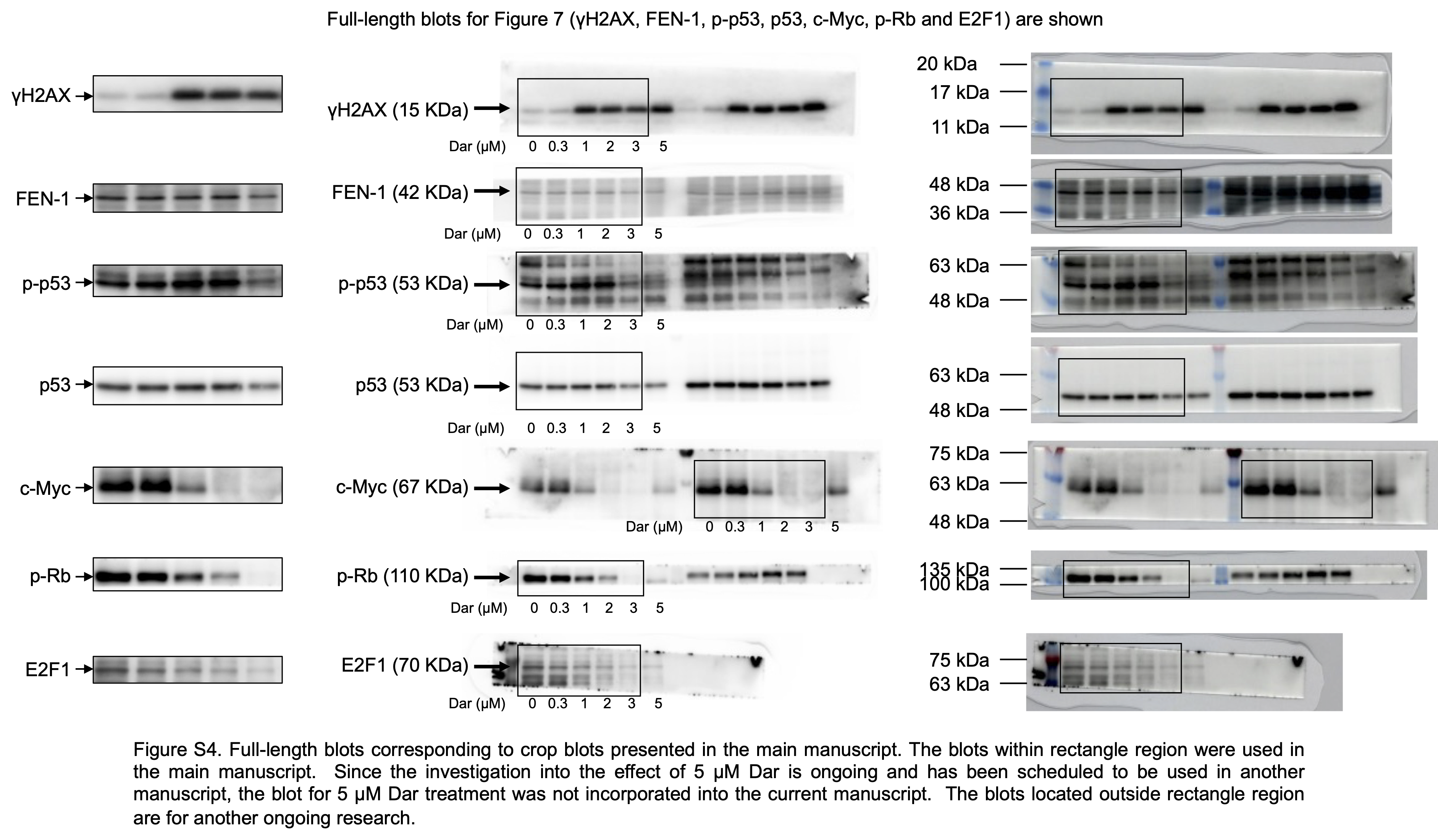

Supplement: Supplementary file 1 [file ijms-24-02282-s001.zip › Figure S4.tiff]

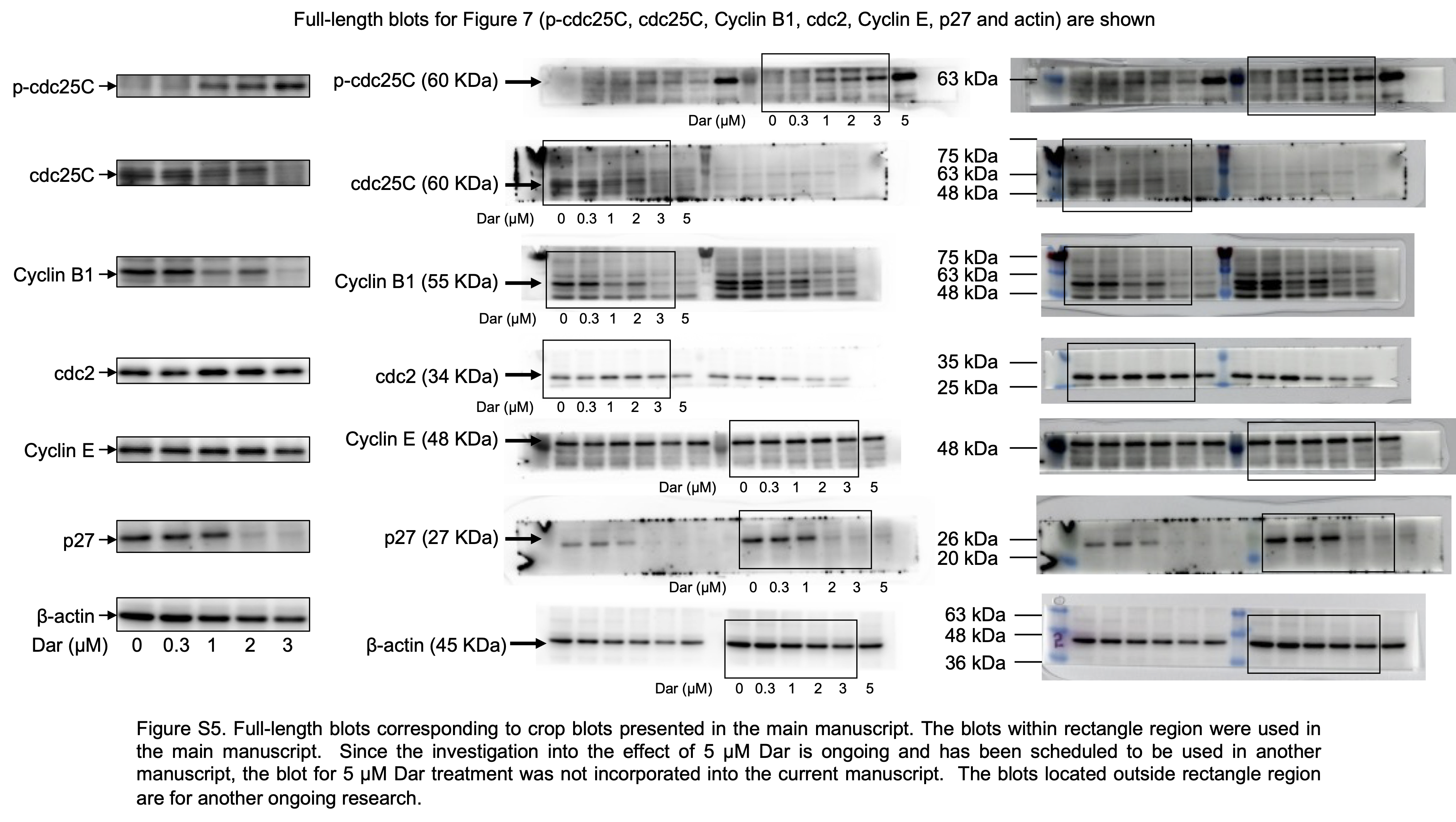

Supplement: Supplementary file 1 [file ijms-24-02282-s001.zip › Figure S5.tiff]

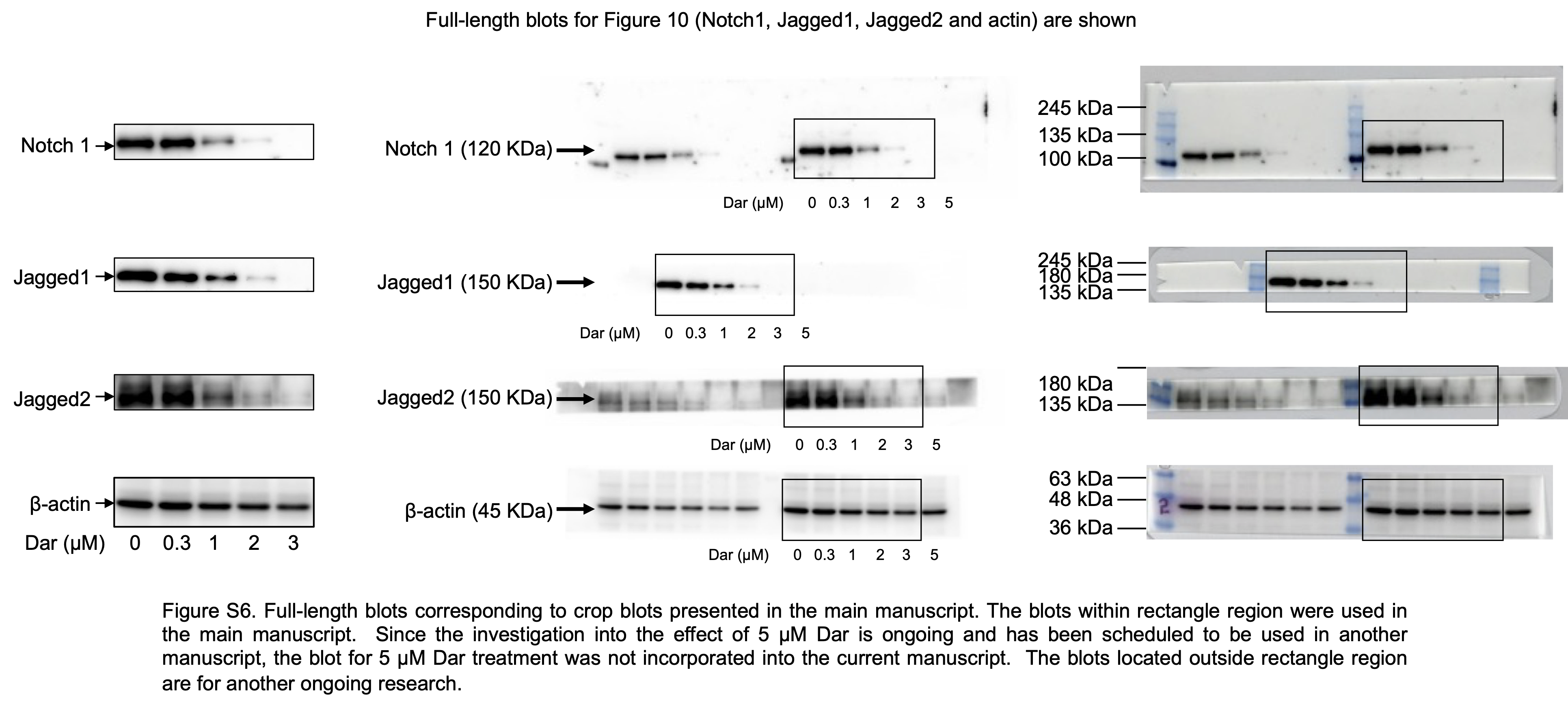

Supplement: Supplementary file 1 [file ijms-24-02282-s001.zip › Figure S6.tiff]
